# Herlyn-Werner-Wunderlich Syndrome: A Case Report

**DOI:** 10.31729/jnma.8096

**Published:** 2023-03-31

**Authors:** Priyanka Vaidya, Pooja Agarwal, Achala Vaidya

**Affiliations:** 1 Department of Obstetrics and Gynaecology, Toowoomba Hospital, Toowoomab, Queensland, Australia; 2 Department of Radiology, Norvic International Hospital, Thapathali, Kathmandu, Nepal; 3 Department of Obstetrics and Gynaecology, Norvic International Hospital, Thapathali, Kathmandu, Nepal

**Keywords:** *case reports*, *mesonephric ducts*, *mullerian ducts*

## Abstract

Herlyn-Werner-Wunderlich syndrome is a rare Mullerian and mesonephric ductal anomaly characterized by a triad of didelphys uterus, obstructed hemivagina, and ipsilateral renal agenesis complex. This entity is also known as obstructed hemivagina and ipsilateral renal anomaly. We present a case of a 24-year-old nulliparous female with Herlyn-Werner-Wunderlich who presented with dysmenorrhea and intermenstrual bleeding. The diagnosis was initially made through ultrasound and confirmed on magnetic resonance imaging. The nonspecific nature of symptoms and variability in presentation depending on the classification and type of Herlyn-Werner-Wunderlich syndrome often leads to misdiagnosis or a delay in diagnosis. Therefore, a high index of suspicion is required.

## INTRODUCTION

The Herlyn-Werner-Wunderluch (HWW) syndrome is a rare congenital syndrome reported in 0.1 to 3.8% of the general population.^1,2^ Herlyn-Werner syndrome (i.e., renal agenesis and ipsilateral blind hemivagina) was initially described in 1971 by Herlyn and Werner. In 1976, Wunderlich described an association of right renal aplasia with a bicornuate uterus and simple vagina in the presence of an isolated hematocervix.^3,4^

HWW generally occurs after menarche during puberty or is identified in young women. The most common presentation includes pelvic pain, dysmenorrhea, irregular menstruation, and palpable abdominal mass secondary to haematocolpos or haematometra.^1,2^ Treatment is surgical, with resection of the septum dividing the two hemivaginas in order to relieve the obstruction.

## CASE REPORT

A 24-year-old nulliparous female presented with a history of severe dysmenorrhea as well as intermittent lower abdominal pain associated with per vaginal spotting and brown discharge.

She had menarche at the age of 12 years and had regular menstrual cycles with normal flow; however, she had severe dysmenorrhea, without menorrhagia. She described intermenstrual spotting and brown discharge between her cycles as well as abnormal vaginal discharge. She had normal bowel and bladder habits. She had no significant past medical or surgical history.

On examination, her abdominal examination was unremarkable. Her external genitalia was examined normally. However, a vaginal examination demonstrated an organized mass on the right fornix.

An initial ultrasound at a regional hospital revealed a bicornuate uterus with an elongated multilocular complex lesion in the right adnexa. A computed tomography (CT) abdomen pelvis was then performed which demonstrated a bicornuate uterus with an elongated tubular cystic lesion in the right adnexa with an incomplete enhancing structure. The differential diagnosis of a right tubo-ovarian mass was proposed based on CT.

Blood was unremarkable with the exception of an elevated C-reactive protein (CRP) of 57.6 mg/dL. Her haemoglobin was 11 g/dL, serology and sexually transmitted infection screen negative, and tumour markers were normal. The patient was then treated as a tubo-ovarian abscess with oral antibiotics and referred to a tertiary centre.

**Figure 1:**
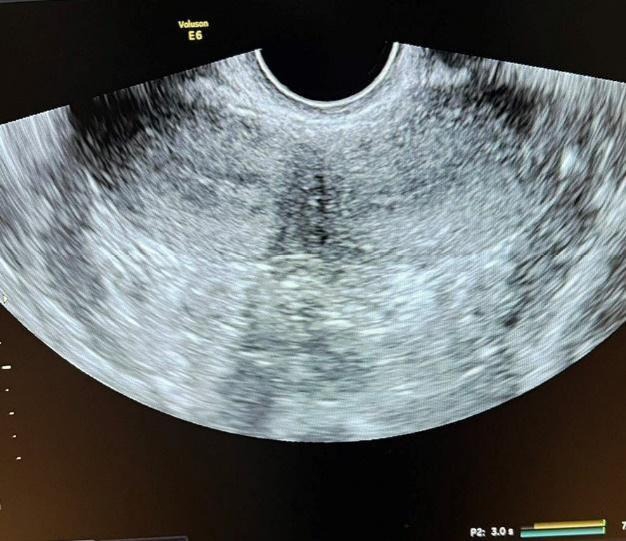
Uterine didelphys on ultrasound.

A tertiary ultrasound done at our hospital revealed: uterine didelphys, an absent right kidney, and a tubular linear structure communicating with the right cervical canal inferiorly (Figure 1).

**Figure 2:**
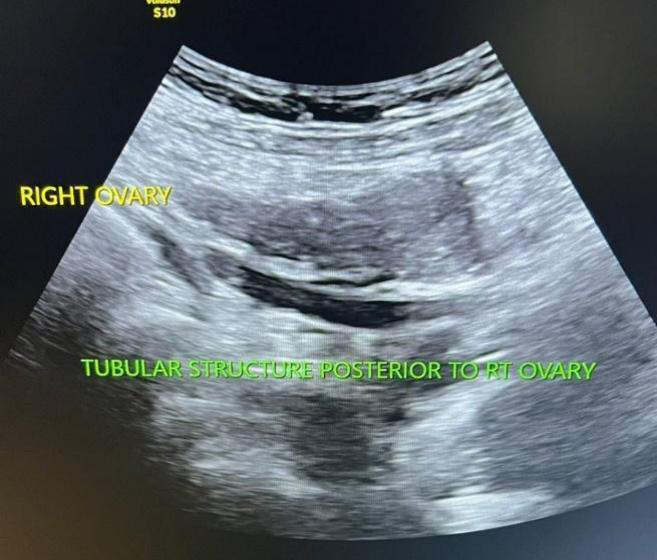
Cystic tubular structure is seen posterior to the uterus opening into the right-sided cervical canal.

Superiorly the tubular structure was blind ending. The right ovary and adnexa were separately visualized from this tubular cystic structure. In view of an absent right kidney, a possible diagnosis of a blind-ending ectopic distal right ureter was considered (Figure 2).

**Figure 3:**
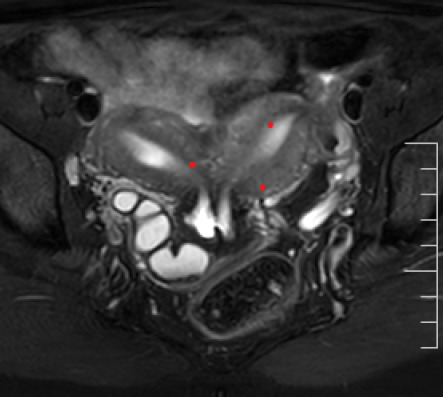
MRI showed uterine didelphys with the duplicated cervix and small communication exists between the two cervix. A cystic tubular structure was noted on the right side.

**Figure 4:**
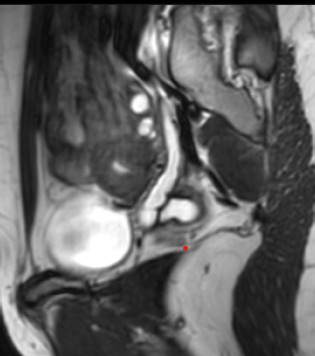
MRI showing a cystic tubular structure which is blind ending superiorly and coursing posterior to cervix, separate from the ovary.

The magnetic resonance imaging (MRI) revealed a didelphys uterus with communicating cervix, an ectopic insertion of the blind-ending distal right ureter into the right cervical canal, and an absent right kidney Hence, a diagnosis of HWW class 2.1 was made (Figure 3,4).

## DISCUSSION

Mullerian duct abnormalities (MDA) are a range of developmental anomalies that result from a nondevelopment, defect in fusion, or regression of fetal development. HWW is characterized as a type III Mullerian duct anomaly accounting for 5% of MDA.^5,6^ The exact aetiology and pathogenesis of the HWW syndrome are still unknown.

The syndrome is classified based on the level of obstruction of the vagina: with class 1 as completely obstructed hemivagina and class 2 as incompletely obstructed hemivagina.^7^ The case described was diagnosed at class 2.1 with partial reabsorption of the vaginal septum, with communication between the two vaginas while the uterus behind the septum remained complexly isolated form the contralateral uterus.

Diagnosis is often made through ultrasonography and confirmed through MRI. Transvaginal ultrasound holds the benefits of being a modality which is accessible, low cost has no radiation and provides quality imaging of the uterus and adnexa. MRI remains the gold standard for diagnosis and classifying the type of HWW. MRI distinguishes the uterine anatomical variants and communications, renal agenesis, and uterine tract variants as well as diagnoses complications like endometriosis. However, MRI is insensitive to detecting vaginal fistulas.^2^

The most common findings are a triad of uterus didelphys, obstructed hemivagina and mesonephric duct anomalies. Typical findings include duplication of the uterus, cervix and vagina as well as unilateral hematocolpos or haematometrocolpos, congenital renal anomalies on the same side of the uterovaginal obstruction, Gartner duct cysts and pelvic endometriosis.^8,9^

When uterovaginal obstruction results in significant dilatation, the imaging differential may include a large adnexal mass such as a cystadenoma, endometrioma, hydrosalpinx/haematosalpinx or a tubo-ovarian complex.^7^

The gold standard for diagnosis and treatment is surgical. Laparoscopy is often performed based on the severity of symptoms and the presence of haematometra or pyometra. Laparoscopy allows therapeutic drainage of the haematometrocolpos, vaginal septotomy or marsupialization of the blind hemivagina.^10^ However, if surgery is not an immediate option for the patient, menstrual suppression with oral contraceptive pills is advised to prevent further accumulation and obstruction.^11^

Early detection and timely treatment of HWW has a good prognostic outcome with preservation of fertility and reduction in complications. The complications associated are often acute, including pyohematocolpos, pyosalpinx, or pelviperitonitis. Long-term complications include chronic pelvic pain, endometriosis and pelvic adhesions from retrograde menstruation.^12,13^ A total of 87% of patients have a successful pregnancy; however, long-term obstetric complications include increased risk of abortion in 23% of patients, premature delivery in 22% of patients or infertility.^10^

Rare complications of adenocarcinoma of the obstructed uterus and cervix or clear cell carcinoma of the obstructed portion of the vagina have also been described.^7^ Complications associated with renal failure are also noted to be high if patients have been diagnosed with renal agenesis; therefore, continuous follow-up for renal function is recommended.^14^

Herlyn-Werner-Wunderlich (HWW) syndrome is a rare congenital anomaly with various vague presentations depending on its type. As demonstrated in our case report, it is difficult to achieve an early accurate diagnosis, as regular menstruation with cyclic dysmenorrhea, vaginal discharge and a raised CRP with a complex right tubo-ovarian complex on imaging can be easily misdiagnosed. We aim to present this case to highlight the entity and the importance of early identification to prevent complications through a high index of clinical suspicion and radiological confirmation.
